# Chronic hyperglycemia is associated with acute kidney injury in patients undergoing CABG surgery – a cohort study

**DOI:** 10.1186/s12872-015-0028-y

**Published:** 2015-05-12

**Authors:** Mehmet Oezkur, Martin Wagner, Dirk Weismann, Jens Holger Krannich, Christoph Schimmer, Christoph Riegler, Victoria Rücker, Rainer Leyh, Peter U. Heuschmann

**Affiliations:** Department of Cardiovascular Surgery, University Hospital Würzburg, Würzburg, Germany; Institute of Clinical Epidemiology and Biometry, University of Würzburg, Würzburg, Germany; Comprehensive Heart Failure Center, University of Würzburg, Würzburg, Germany; Department of Internal Medicine I, Division of Nephrology, University Hospital Würzburg, Würzburg, Germany; Department of Internal Medicine I, Endocrine and Diabetes Unit, University Hospital Würzburg, Würzburg, Germany; Clinical Trial Center Würzburg, University Hospital Würzburg, Würzburg, Germany

## Abstract

**Background:**

Chronic hyperglycemia (CHG) with HbA1c as an indicator affects postoperative mortality and morbidity after coronary artery bypass grafting surgery (CABG). Acute kidney injury (AKI) is one of the frequent postoperative complications after CABG impacting short-and long-term outcomes. We investigated the association between CHG and postoperative incidence of AKI in CABG patients with and without history of diabetes mellitus (DM).

**Methods:**

This cohort study consecutively enrolled patients undergoing CABG in 2009 at the department for cardiovascular surgery. CHG was defined as HbA1c ≥ 6.0 %. Patients with advanced chronic kidney disease (CKD) were excluded. The incidence of postoperative AKI and its association with CHG was analyzed by univariate and multivariate logistic regression modeling.

**Results:**

Three-hundred-seven patients were analyzed. The incidence of AKI was 48.2 %. Patients with CHG (n = 165) were more likely to be female and had greater waist circumference as well as other comorbid conditions, such as smoking, history of DM, CKD, hypertension, pulmonary hypertension, and chronic obstructive pulmonary disease (all *p* ≤ 0.05). Preoperative eGFR, atrial fibrillation (AF), history of DM and CHG were associated with an increased risk of postoperative AKI in univariate analyses. In multivariate modelling, history of DM as well as preoperative eGFR and AF lost significance, while age, CHG and prolonged OP duration (*p* < 0.05) were independently associated with postoperative AKI.

**Conclusions:**

Our results suggest that CHG defined on a single measurement of HbA1c ≥ 6.0 % was associated with the incidence of AKI after CABG. This finding might implicate that treatment decisions, including the selection of operative strategies, could be based on HbA1c measurement rather than on a recorded history of diabetes.

## Background

Acute kidney injury (AKI) is a frequent postoperative complication with an incidence ranging from 20 to 40 % in patients undergoing cardiac surgery [1–6]. Even slight changes in creatinine-levels, *e.g*. by 0.3 mg/dl (AKI Stage I [7]), are associated with impaired long-term outcome, such as risk of mortality or development or progression of chronic kidney disease (CKD), as multiple studies have indicated in CABG patients [4–6, 8]. Age, gender, preoperative creatinine, diabetes mellitus (DM), and metabolic syndrome, but also urgent operations and operation duration have been discussed as potential risk factors for AKI [1, 9–13]. Both, chronic and acute hyperglycemias are associated with cardiac dysfunction, susceptibility for infections and endothelial dysfunction [14–18]. These factors are known risk factors of perioperative morbidity and mortality after CABG surgery, in particular of renal dysfunction [1, 9, 11–13, 19–21].

HbA1c is an established parameter for evaluating diabetic control and chronic hyperglycemia (CHG) in patients with DM [22]. Elevated HbA1c levels have been implemented in recent guidelines [22] and can now be used for diagnosis of diabetes. Furthermore, HbA1c has been identified as an important marker for insulin resistance, risk for acute hyperglycemia and endothelial dysfunction and atherosclerosis in diabetic and non-diabetic patients [14, 16].

A history of diabetes at baseline clinical assessment is frequently used as a marker of cardiovascular risk of individual patients after cardiac surgery [19–21]. However, information on history of diabetes mellitus is often based on self-reports of patients or from the patient’s records and does not reflect current glucose control. Furthermore, even patients without the formal criteria for DM can suffer from hyperglycemia due to pathological glycemic control or pre-DM [22]. It is unknown whether preoperative CHG is associated with the risk of AKI after cardiac surgery.

In the current study, we aimed to investigate the association between CHG and postoperative incidence of AKI in a cohort of CABG patients, independent from potential confounders including history of diabetes.

## Methods

The study protocol of this prospective cohort study was approved by the Ethics Committee at the University Hospital of Würzburg. All patients who received cardiac surgery between January 1^st^ and December 31^st^ 2009 were screened for participation. Patients were included if they underwent CABG surgery or combination procedures including CABG. Further inclusion criteria were elective or urgent (start of operation > 6 h after admission) CABG surgery, and HbA1c-level measured on admission. Exclusion criteria were operations in which CABG was not planned preoperatively, age < 18 years, pregnancy and lactation, advanced stages of chronic kidney disease (CKD) with a eGFR <30 ml/min/1.73 m^2^ according to the creatinine-based CKD-EPI equation, acute infection (C-reactive protein >4.0 mg/dl, procalcitonin >1.0 μg/l), high-urgent or salvage surgery (preoperative resuscitation, i.v. catecholamine therapy started preoperatively, assist device, operation < 6 h after admission).

The primary endpoint of the present study was defined as the postoperative incidence of any stage of AKI, according to recent KDIGO guideline recommendations [23], similar to the AKIN - and STS criteria [24]. Thus, even a slight creatinine level rise of 0.3 mg/dl compared to baseline within 48 hrs after surgery or Creatinine 1.5 to 1.9-times baseline (stage I) was defined as AKI. Creatinine values of blood-samples drawn at hospital admission were considered as baseline. More severe stages of AKI were a rise in Creatinine 2.0 to 2.9-times baseline (stage II) and ≥3-times baseline, Creatinine ≥4 mg/dl or the need of renal replacement therapy (stage III).

Standard demographic data of patients were collected and comorbidities such as a history of diabetes, hypertension, pulmonary hypertension and smoking were taken from the patient’s chart and personal interview. Furthermore, intraoperative data (e.g., operation time, paracorporal bypass time, aortic cross clamping time, etc.) and standard laboratory results of clinical routine including HbA1c measurement at admission were collected. Preoperative chronic hyperglycemia was defined as an HbA1c ≥ 6.0 % on admission [17, 18, 22]. Postoperative serum glucose (SG) was taken on admission to the ICU after surgery. Postoperative hyperglycemia was defined as fastening serum glucose of ≥126 mg/dl during the postoperative course.

Intraoperative data about relevant time-points were taken from the digital protocol of the heart-lung machine. Metformin was stopped 24 hours before admission. For the purposes of the study, chronic kidney disease (CKD) was defined as eGFR <60 ml/min.

### Statistical analyses

Patient demographics are presented as mean and standard deviations, medians and interquartile ranges and number of observations with proportions (%), as appropriate. Differences across group were assessed by t-test, Mann-Whitney-U-Test/ and χ^2^-test/Fisher’s exact test, respectively. Determinants of the postoperative incidence of AKI which were significant in the univariate analyses (age, history of DM, HbA1c, eGFR, AF, OP duration, COPD, metabolic syndrome and sex) were investigated by logistic regression analysis. In a sensitivity analysis, we tested the robustness of the results in a propensity score approach: age, history of DM, HbA1c, eGFR, AF, OP duration, COPD, metabolic syndrome and sex were included to assess the probability of having a HBA1c level >/< 6.0 % by multivariate logistic regression modelling. These results were included in the multivariate regression analysis, modelling AKI as a covariate.

Results of regression analyses are presented as Odds Ratio with respective 95 % confidence interval (CI). Two-sided *p*-values of ≤0.05 were considered as statistically significant. Statistical analyses were performed using SPSS Version 21.

## Results

### Patient population

In 2009, a total of n = 928 adult patients underwent cardiac surgery at the department of cardiovascular surgery at the University Hospital of Würzburg. Of these, 478 received elective or urgent CABG surgery. Thirty-nine patients were excluded because eGFR was less than 30 ml/min, as well as were two patients with acute infection and 75 high urgent/salvage surgery patients. Furthermore, no HbA1c measurement within the clinical routine was available for 55 patients. Therefore the study population consisted of 307 patients.

Patient characteristics of the entire cohort and stratified according to the established HbA1c cutoff <6.0 % and ≥ 6.0 %, respectively) [25], are displayed in Table 1. Patients had a median age of 69 years, 73.9 % were male A history of diabetes was observed in 35 % of patients. Metabolic syndrome according to the definition of WHO (considering DM, hypertension, dyslipidemia and BMI) was detected in 254 (82.3 %) of the patients. Thirty-three (10.7 %) patients were operated with off pump coronary artery bypass (OPCAB), and 2.6 % (n = 8) had an on-pump beating heart procedure. Patients with higher HbA1c levels were more likely to be female and with greater waist circumference. Furthermore, comorbid conditions, such as smoking, history of DM, hypertension, pulmonary hypertension and CKD, were detected more frequently as compared to patients with lower HbA1c levels (all p ≤ 0.05).

**Table 1:** Patients characteristics

Variable	All patients (*n* = 307)	HbA1c < 6.0 % (*n* = 142)	HbA1c ≥ 6.0 % (*n* = 165)	*P*-value
Age, years	69(60-75)	67(57-74)	70(63-75)	0.008
Male, n (%)	227(73.9)	116 (81.7)	111 (67.3)	0.004
Smoking, n (%)	121(39.4)	47(33.1)	74(44.8)	0.046
LV-EF, %	55(45-63)	55(49-65)	55(45-61)	0.048
Waist circumference, cm	103(±11)	102(±10)	105(±12)	0.047
BMI, kg/m^2^	28(25-30)	27(25-29)	28(96-113)	0.087
Urgent surgery, n (%)	134(42.6)	70(49.3)	64(38.8)	0.066
MI within the last 48 h, n (%)	25(8.1)	15(10.6)	10(6.1)	0.19
Previous cardiac surgery, n (%)	13(4.2)	6(4.2)	7(4.2)	1.00
Metabolic syndrome, n (%)	254(82.7)	107(75.4)	147(89.1)	0.002
COPD, n (%)	57(18.6)	19(13.4)	38(23)	0.039
History of DM, n (%)	106(34.5)	15(10.6)	91(55.2)	<0.001
HbA1c, %	6.0(5.7-6.5)	5.7(5.5-5.8)	6.5(6.2-7.0)	<0.001
FSG, mg/dl	105(97-123)	101(95-106)	116(102-138)	<0.001
Postoperative SG, mg/dl	143(120-179)	136(117-165)	154(125-188)	<0.001
Postop. hyperglycemia, n (%)	209(68.1)	88(42.1)	121(57.9)	0.037
Hypercholesterolemia, n (%)	181(59.0)	80(56.3)	101(61.2)	0.42
Hypertension, n (%)	267(87.0)	122(85.9)	145(87.9)	0.62
Pulmonary Hypertension, n (%)	54(11.3)	16(11.3)	38(23.0)	0.007
AF, n (%)	28(9.1)	8(5.6)	29(12.1)	0.072
TG, mg/dl	128(95-184)	122(89-163)	137(101-205)	0.016
HDL, mg/dl	43(37-53)	44(38-53)	42(35-54)	0.47
LDL, mg/dl	104(82-128)	109(90-135)	98(73-119)	<0.001
Preoperative creatinine, mg/dl	1.0(0.8-1.2)	1.0(0.8-1.2)	1.0(0.8-1.2)	0.55
Preoperative eGFR_CKD-EPI_, ml/min/1.73 m^2^	74(59-89)	78(62-100)	72(56-86)	0.010
CKD, n (%)	82(26.7)	29(20.4)	53(32.1)	0.028
OP Duration, min	245(219-295)	240(218-291)	250(219-299)	0.91
CPB Time, min	107(88-143)	105(86-137)	115(88-151)	0.07
X-Clamp Time, min	81(61-104)	78(57-95)	83(63-110)	0.05

Data are mean (±SD), number (%) or median (IQR); *LV-EF*: left ventricular ejection fraction; *BMI*: body mass index; *MI*: myocardial infarction; *COPD*: chronic obstructive pulmonary disease; *DM*: diabetes mellitus; *FSG*: fastening serum glucose; *AF*: atrial fibrillation; *HDL*: high density lipoprotein, *LDL*: low density lipoprotein, *eGFR*: estimated glomerular filtration rate; *CKD*: chronic kidney disease; *X-clamp*: aortic cross-clamp; *AKI*: acute kidney injury

All 106 patients with a history of diabetes were treated with some kind of diabetes therapy, with 34 % being on insulin therapy. In only 15 diabetic patients, glucose metabolism was strictly controlled (HbA1c <6.0 %) with a median FSG of 105 mg/dl. Ninety-one diabetic patients had HbA1c >6.0 % (median FSG 140 mg/dl) of which 65 had HbA1c levels greater than 6.5 %. None of the patients without a recorded history of diabetes was treated with oral antidiabetics or insulin therapy. However, although median FSG in this group was 113 (104 – 123) mg/dl, HbA1c levels >6.0 % were observed in 74 patients, suggesting CHG.

A history of DM was significantly related to postoperative hyperglycemia regardless of HbA1c (OR 1.46 (1.00 – 2.14), *p* = 0,028), whereas acute postoperative hyperglycemia was not associated with the incidence of AKI (OR 1.00 (1.00 – 1.01), *p* = 0.96).

#### Postoperative incidence of AKI

A total of n = 148 patients (49.3 %) experienced AKI (Table 2). Most of the episodes were stage I (n = 125, 41.1 %) with only a slight increase of serum creatinine. In 23 patients (7.5 %) a more severe increase in creatinine was observed and two patients required hemodialysis treatment. Patients with HbA1c ≥ 6.0 % were more likely to develop AKI across all stages, as compared to patients with HbA1c < 6.0 % (Table 2).

**Table 2:** Outcomes

Variable	All patients (*n* = 307)	HbA1c < 6.0 % (*n* = 142)	HbA1c ≥ 6.0 % (*n* = 165)	*P*-value
AKI, n (%)	148(49.3)	57(41.0)	91(56.5)	0.008
AKI Stage I, n (%)	125(41.1)	49(34.5)	76(46.9)	0.005
AKI Stage II, n (%)	22(7.2)	8(5.6)	14(8.6)	0.028
AKI Stage III, n (%)	1(0.3)	0(0)	1(0.6)	0.31
Creatinine Peak, mg/dl	1.3(1.1-1.7)	1.3(1.0-1.6)	1.4(1.1-1.8)	0.014
Mortality, n (%)	10(3.3)	0	10(6.1)	0.002

Data are number (%) or median (IQR) *AKI*: acute kidney injury

#### Risk factors of AKI

In univariate logistic regression analysis (Table 3), a history of diabetes as well as elevated levels of HbA1c were associated with the incidence of postoperative AKI. Furthermore, older age, impaired kidney function, preoperative atrial fibrillation and prolonged operation time were significantly associated with an increased risk of AKI (all *p* < 0.05). Male gender, FSG and COPD approached significance (*p* < 0.10). In a sensitivity analysis, in which we included the propensity of each patient as presenting with a HbA1c level of >6.0 % in the multivariate regression model for AKI, the results remained largely unchanged, with a HbA1c level >6.0 % being related to a higher risk of AKI (OR 1.71, 95 % CI 0.90-3.26, *p* = 0.10), but this association lost significance (detailed data not shown).

**Table 3:** Determinants of postoperative AKI (logistic regression analysis)

Variable	Univariate	Multivariate	
		OR (CI95 %)	*p*-value
Age	1.08 (1.05-1.11)	1.07 (1.04-1.10)	<0.001
History of DM	1.85 (1.14-3.00)	1.30 (0.72-2.37)	0.39
HbA1c ≥ 6.0 %	1.87 (1.18-2.96)	1.65 (1.00-2.71)	0.049
Preop. eGFR (ml/min)	0.82 (0.72-0.93)	1.00 (0.98-1.01)	0.73
AF	3.05 (1.24-7.49)	2.21 (0.86-5.67)	0.098
OP Duration (min)	1.07 (1.03-1.11)	1.05 (1.01-1.10)	0.013
Sex	1.45 (0.87-2.49)	1.04 (0.58-1.88)	0.89
FSG	1.01 (1.00-1.01)	1.00 (0.99-1.01)	0.73
COPD	1.76 (0.98-3.19)	1.44 (0.76-2.74)	0.26
Post. OP SG	1.00 (1.00-1.01)		
Post. OP HG	1.05 (0.83-1.34)		
Pulm. Hyper.	1.25 (0.68-2.30)	-	-
Smoking	1.02 (0.64-1.62)	-	-
Urgent surgery	0.85 (0.54-1.33)	-	-
BMI	1.00 (0.96-1.07)	-	-

Data are OR (95 % CI); *eGFR*: estimated glomerular filtration rate; *AF*: atrial fibrillation; *FSG*: fastening serum glucose; *BMI*: body mass index*;* final model based on backward stepwise selection; last OR before elimination shown

#### Mortality

During the hospital stay (median 9 days, IQR 8-12), ten patients died (3.3 %), with a median time to death of 6 days (IQR 1-21). Mortality was not significantly associated with the incidence of AKI (OR: 1.62; 95 % CI: 0.40 – 6.67; *p* = 0.72). However, all patients who died had HbA1c ≥ 6.0 % (*p* = 0.002) as compared to survivors. Further determinants of mortality in univariate analyses were older age, impaired LV-EF, preoperative eGFR, female gender and pulmonary hypertension (*p* < 0.05).

## Discussion

The results of our study suggest that aside from older age and prolonged OP duration, CHG, i.e. elevated HbA1c levels were associated with the postoperative incidence of AKI after CABG surgery. This association was independent from of a history of diabetes, which was only associated with the risk of AKI in univariate analyses. Furthermore, preoperative kidney function lost its univariate association with AKI in multivariate modelling. Even controlling for a multitude of clinical factors employing a propensity score approach suggested an important role of CHG, although this association failed to reach formal statistical significance.

This is of particular interest, as usually a history of diabetes is used in daily clinical practice for clinical decision making including the choice of specific operation strategies as harvesting both mammarial arteries or the extend of the operation. The easy-to-use laboratory marker HbA1c (as compared to the time consuming oGTT) not only provides additional information on the glycemic state of the patient over the last weeks, it is also an indicator for insulin resistance providing information on perioperative incidences of hyperglycemia [14]. Markers of impaired glucose metabolism such as elevated HbA1c levels, history of DM and high levels of FSG were associated with an increased risk of AKI after CABG surgery in univariate analyses. Postoperative glucose control is reported as a risk factor for renal impairment after cardiac surgery [14]. In our cohort, we could not find any strong relation of postoperative hyperglycemia and the development of AKI. The overall incidence of AKI (48 %) was higher than described in recent literature (20 to 40 %) [3, 4, 6, 8, 13]. Compared to these previous studies we used the rigorous definition of AKI as recommended by KDIGO guidelines 2012, of even a slight increase of serum creatinine by 0.3 mg/dl [23]. This definition has also been implements in the recommendations of the STS [24]; although the changes in creatinine are considerably minor, it has been shown that they carry important information predictive for the development of long-term outcome, including aggravated risk of the development of CKD as well as cardiac dysfunction [5, 6]. The preoperative fastening status might have caused hypovolemia which further could have sensitized the kidney for intraoperative stress and thus the development of AKI. However, only few patients experienced severe AKI and only two needed renal replacement therapy. After multivariate modelling, the association of HbA1c ≥ 6.0 % with AKI weakened but stayed statistically significant, suggesting a nearly 1.7-fold increased risk for patients with CHG independent from the history of DM.

In-hospital mortality was comparable to other studies (3.3 %) and was not significantly associated with AKI. In contrast, all patients who died had HbA1c ≥ 6.0 % at admission, as well as they were older, more likely to be of male gender, with previous cardiac surgery and prolonged operation duration in univariate analyses. The overall considerably low mortality-rate did not allow a multivariate analysis of mortality. Our results suggest that the strict criteria for AKI might not have a significant impact on the short-term clinical outcome of patients undergoing CABG surgery, however, due to limited sample size this interpretation needs to be taken with caution.

We also found operation time with respective prolonged duration of CPB and X-Clamp time as another strong and independent risk factor for the incidence of postoperative AKI. Per minute prolonged operation time (including CBP and X-clamp time), the risk of AKI increased by 1 %. Prolonged OP duration potentiates inflammatory processes and the formation of oxidative stress metabolites which are known to affect kidney function, and promote the development of AKI [9, 10, 12]. In our cohort, patients with OPCAB operations showed a high risk for AKI. Most likely treatment bias can be considered as the key issue for that finding – patients were operated OPCAB or beating-heart on-pump when X-clamping or CPB was impossible due to severe calcification of the ascending aorta and aortic arch. We assume that those patients had severe general calcification of their arteries, which might also affect renal arteries and the glomerular convolute. This is also supported by the fact that patients operated OPCAB or beating-heart on-pump had a higher prevalence of hemodynamic stenosis of A. carotis interna (data not shown).

Metabolic syndrome (MS) has been described as a risk factor for AKI in CABG patients [1]. Only 17.7 % of our cohort did not fulfill the MS criteria according to the definition of WHO. We could not show a statistically signification association of MS and AKI potentially due to the relatively limited number of observations. DM is one of the components of MS and can therefore be seen as an important confounder for the association between MS and AKI described in other studies [26].

We are aware of several limitations of the current study. HbA1c values were not kept blinded to the treating physicians, therefore, specific treatment decisions might have been based on the knowledge of certain HbA1c values (treatment bias). Furthermore, the size of our study sample is relatively limited, although a quite large number of events was observed. Therefore we were not able to assure sufficient statistical power for all our hypotheses as well as for the inclusion of other known risk factors of AKI (e.g. volume management during CPB and medication) in our multivariate models. We refrained from testing too many variables in these models, as we wanted to avoid finding associations by chance (overfitting the model), however, we might have missed “true” associations by chance as well.

## Conclusion

Our data suggest that CHG based on a single measurement of HbA1c ≥ 6.0 %, could represent a strong determinant of AKI and mortality after CABG surgery, independent from a recorded history of DM. This finding might implicate that treatment decisions, including the selection of operative strategies, could be based on HbA1c measurement rather than on a recorded history of diabetes. This applies to patients with treated diabetes as well as to those without. Further prospective studies are needed to confirm our findings and to investigate how treatment decisions based on HbA1c levels independent from or in addition to the knowledge of diabetic status of the patient might improve clinical outcomes after cardiac surgery.

### Consent

Written informed consent was obtained from the patient for the publication of this report and any accompanying images.

## Abbreviations

AKI: Acute kidney injury
BMI: Body mass index
AKIN: Acute kidney injury network
CABG: Coronary artery bypass grafting
CAD: Coronary artery disease
CHG: Chronic hyperglycemia
CKD: Chronic kidney disease
eGFR: Estimated glomerular filtration rate
FSG: Fastening serum glucose
KDIGO: Kidney disease improving global outcome
LV-EF: Left ventricular ejection fraction
MAP: Mean atrial pressure
MS: Metabolic syndrome
OPCAB: Off pump coronary artery bypass
SG: Serum glucose
WHO: World Health Organization
